# A comparison of carbon ions versus protons for integrated mode ion imaging

**DOI:** 10.1002/mp.17645

**Published:** 2025-02-04

**Authors:** Mikaël Simard, Ryan Fullarton, Lennart Volz, Christoph Schuy, Daniel G. Robertson, Allison Toltz, Colin Baker, Sam Beddar, Christian Graeff, Charles‐Antoine Collins Fekete

**Affiliations:** ^1^ Department of Medical Physics and Biomedical Engineering University College London London UK; ^2^ Biophysics GSI Helmholtz Centre for Heavy Ion Research GmbH Darmstadt Germany; ^3^ Division of Medical Physics Department of Radiation Oncology Mayo Clinic Arizona Phoenix Arizona USA; ^4^ Department of Radiotherapy Physics University College London Hospital NHS Foundation Trust London UK; ^5^ UTHealth Graduate School of Biomedical Sciences The University of Texas MD Anderson Houston Texas USA

**Keywords:** image‐guided radiotherapy, integrated mode, ion beam therapy, ion imaging, ion radiography

## Abstract

**Background:**

Incorporating image guidance into ion beam therapy is critical for minimizing beam range uncertainties and realizing the modality's potential. One promising avenue for image guidance is to capture transmission ion radiographs (iRads) before and/or during treatment. iRad image quality is typically maximized using a single‐event imaging system, which involves tracking individual ions, albeit the approach is generally not suited to clinical beam settings. An alternative faster and clinically compatible method is integrated mode imaging, where individual pencil beam data is acquired, rather than single ion data. To evaluate the usefulness of transmission ion imaging for image guidance, it is crucial to evaluate the image quality of integrated mode iRad systems.

**Purpose:**

We report extensive image quality metrics of integrated mode carbon ion radiographs (cRads) and compare them with proton radiographs (pRads).

**Methods:**

iRads were obtained at the Marburg Ion Beam Therapy Center using a plastic volumetric scintillator equipped with CCD cameras. The detector captures orthogonal views of the 3D energy deposition in the scintillator from individual pencil beams. Four phantoms were scanned using a $15\times 15\;{\mathrm{cm}}^{2}$ field of view and a beam spacing of 1 mm. First, 9 tissue‐substitute inserts were used to evaluate water equivalent thickness (WET) accuracy. Radiographs of those inserts were reconstructed for beam spacings ranging from 1 to 7 mm to evaluate the impact of spacing on quantitative accuracy. For spatial resolution, custom 3D printed line pair (lp) modules ranging from 0.5 to 10 lp/cm were scanned. To evaluate low contrast detectability, a custom 3D printed low contrast module consisting of 20 holes with depths ranging from 1 to 8 mm and diameters from 1 to 10 mm was scanned. iRads of an anthropomorphic head phantom were also obtained.

**Results:**

Spatial resolution and low contrast detection are systematically improved for cRads compared to pRads. Image resolution was 3.7 lp/cm for cRads and 1.7 lp/cm for pRads in the center of the field of view. Spatial resolution was found to vary with the object's location in the field of view. While pRads could mostly resolve low contrast holes of 10 mm in diameter, cRads could resolve holes of up in 4 mm diameter. WET accuracy is similar for both ion species, with a root mean squared error of approximately 1 mm. WET accuracy was stable (maximum of 0.1 mm increase) across beam spacings, although important under‐sampling artifacts were observed for iRads reconstructed using large beam spacings, especially for cRads. iRads of the anthropomorphic head phantom showed improved apparent contrast using cRads, especially to identify bony structures.

**Conclusions:**

This work is the first investigation of image quality metrics such as spatial resolution and low contrast detectability for integrated mode cRads, with a full comparison with pRads. Enhanced image quality is obtained with cRads compared to pRads, although pRads still maintain high WET accuracy and deliver image quality within acceptable bounds.

## 1 INTRODUCTION

Proton beam therapy promises many advantages over conventional radiotherapy, although uncertainties in beam range estimation pose limitations on the achievable dose conformity. Using the proton beam in a transmission imaging setup to acquire proton radiographs (pRads) is an avenue of interest to limit uncertainties in proton beam therapy. The main advantage of pRads lies in generating line integrals of the relative proton stopping power, referred to as the water equivalent thickness (WET). In terms of applications, pRads may be used for indirect in‐vivo range verification, low‐dose image guidance at the treatment site, or for managing patient positioning errors.^1^, ^2^, ^3^ Beyond proton therapy, heavier ion therapy, such as carbon ion therapy, offers therapeutic benefits for radioresistant lesions due to their increased relative biological effectiveness.^4^, ^5^, ^6^ In that context, there is equally strong interest in exploring the use of ion radiographs (iRads), notably carbon ion radiographs (cRads), to manage ion beam range uncertainties.^1^

Evaluating the potential benefits of iRads for ion beam radiotherapy necessitates a careful evaluation of the image quality for such systems. State‐of‐the‐art image quality for iRads is typically obtained with single‐event imaging systems that track the trajectory and energy deposition of each ion. Previous studies investigated the spatial resolution and contrast‐to‐noise ratio for helium ions radiographs (HeRads) against pRads,^7^ or the spatial resolution of HeRads alone.^8^ However, single‐event imaging is currently too slow to be used practically for image guidance, is generally incompatible with clinical ion beam settings as it requires lower particle fluxes for data acquisition, and the imposed detector requirements can only be met by complex, fairly expensive detector setups.^9^

A more accessible approach to generate iRads is through integrated mode imaging, where data is acquired on an individual pencil beam basis, and each pencil beam data is analyzed independently. While compatible with clinical beam settings, this approach typically suffers from lower image quality due to the stronger impact of multiple Coulomb scattering (MCS) on the measured datasets.^10^ There have been initial investigations of image quality for integrated mode iRads, including a qualitative assessment of cRads on an anthropomorphic head phantom,^11^ a comparison of pRads, HeRads, and cRads for quantitative WET accuracy^12^ or for patient‐specific CT calibration.^13^ While of interest, those studies do not provide a comprehensive evaluation of conventional image quality metrics such as spatial resolution and detection of low contrast objects.

This work therefore aims to report a more extensive evaluation of image quality metrics for integrated mode iRads. Specifically, we compare the image quality of pRads against cRads acquired using a detector consisting of a volumetric plastic scintillator with CCD cameras.^14^, ^15^ We report results on low contrast object detection, spatial resolution for various locations in the imaging field of view (FOV), WET accuracy on tissue substitute inserts and a more general, qualitative image quality assessment for an anthropomorphic head phantom.

## 2 METHODS

### 2.1 Data acquisition

**FIGURE 1:**
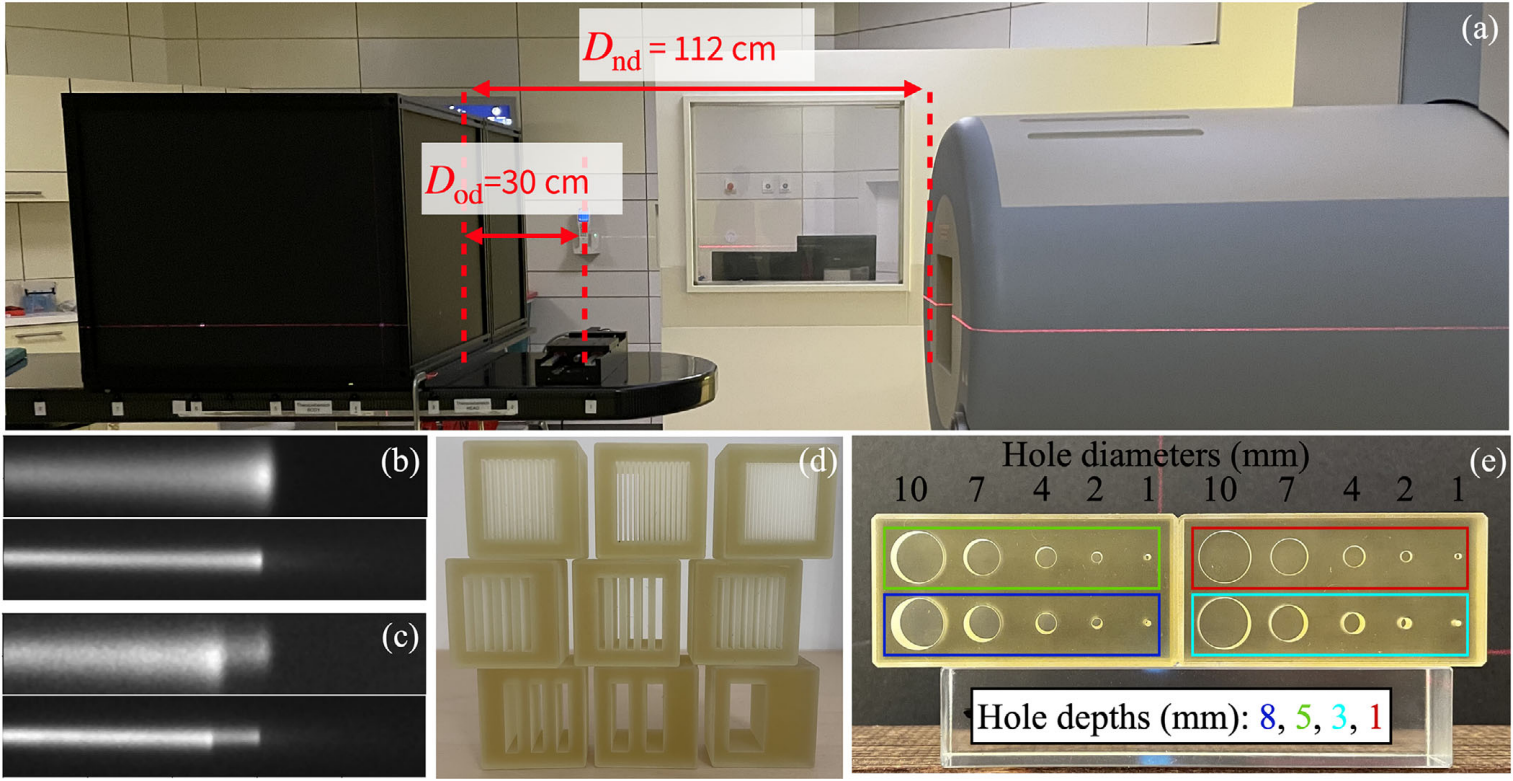
(a) Photograph of the experimental setup at the Marburg Ion Therapy Center. The distance between the nozzle and the detector $D_{\mathrm{nd}}$ is 112 cm. The detector is defined as the surface of the scintillator block facing the nozzle, which is also set at the isocenter. The surface of scintillator is 1 cm from the edge of the black encasing. The distance between the object and the detector $D_{\mathrm{od}}$ is 30 cm; samples are placed where the black sample holder resides on the couch. (b) Example of a dose deposition pattern in the scintillator for a proton beam (top) and a carbon ion beam (bottom) after traversing an homogeneous material. (c) similar to (b), but after propagation through a heterogeneous region, showing range mixing effects. The remaining figures show the phantoms used to evaluate (d) spatial resolution and (e) low contrast detectability.

iRads were acquired at the Marburg Ion Beam Therapy Center (MIT) (Marburg, Germany). The detector was a plastic volumetric scintillator equipped with three CCD cameras capturing orthogonal views of the 3D energy deposition in the scintillator, and covered a $20\times 20\;{\mathrm{cm}}^{2}$ FOV. Additional details on the detector can be found in previous work.^14^, ^15^ A photograph of the experimental setup and relevant distances is shown in Figure 1a; the object to nozzle distance represents a clinically realistic scenario.

cRads were acquired at an energy of 344.62 MeV/u and a spot size (full width at half maximum, [FWHM]) of 5.2 mm at the isocentre, while pRads were obtained at 180.06 MeV and a spot size of 9.4 mm (FWHM). Energies were selected to obtain a similar range for carbon ion and proton beams. All objects were scanned using a pencil beam scanning approach with a 15.1 × 15.1 ${\mathrm{cm}}^{2}$ field size and a beam spacing of 1 mm. For every camera and scanned object, this created a total of 22 801 images, each representing the dose deposition of an individual pencil beam. Example raw data obtained with one camera showing the lateral view of the beam are provided in figure 1b and 1c.

To evaluate image resolution, nine custom line pair (lp) modules were 3D printed, as shown in Figure 1d. Modules were printed on a 3DSystems ProJet MJP 2500 Plus 3D‐printer using VisiJet M2S‐HT250 as printing material and VisiJet M2 SUP as support material. The printing material had a water equivalent density of 1.162 ${\text{g/cm}}^{3}$, which was validated in prior experiments using proton beams. Each lp module was a $3\times 3\times 3\;{\mathrm{cm}}^{3}$ cube representing the following spatial frequencies: 0.5, 1, 1.5, 2, 2.5, 3, 5, 7, and 10 lp/cm. The modules were scanned three at a time using a custom 3D printed module holder (see the inset picture of Figure 3a) such that one insert was in the center of the FOV, another insert was on the right edge of the FOV and the last insert was on the top left corner of the FOV. Each module was scanned in all FOV positions to evaluate the heterogeneity of spatial resolution in the imaging FOV. A 10 cm slab of PMMA was placed proximal to the modules in beam direction to produce realistic scattering conditions.

**FIGURE 2:**
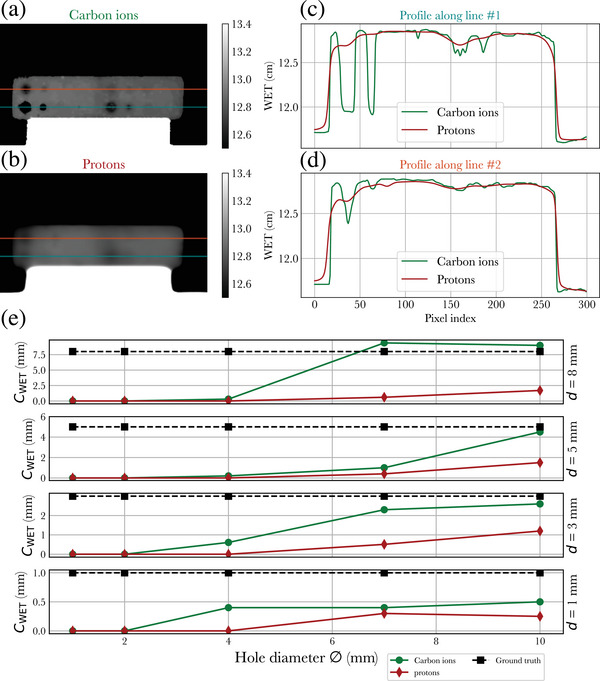
Detection of low contrast objects. (a) and (b) respectively illustrate the reconstructed WET maps of the low contrast phantom (shown in Figure 1e) using carbon ions and protons. The two profile lines shown in the radiographs are reproduced in plots (c) and (d), respectively, the bottom and top lines. (e) illustrates the calculated contrast $C_{\mathrm{WET}}$ as a function of hole diameter ⌀; each subplot is for a fixed hole depth d. The theoretical $C_{\mathrm{WET}}$ is reported as a dashed black line and is equal to the hole depth d. WET, water equivalent thickness.

**FIGURE 3:**
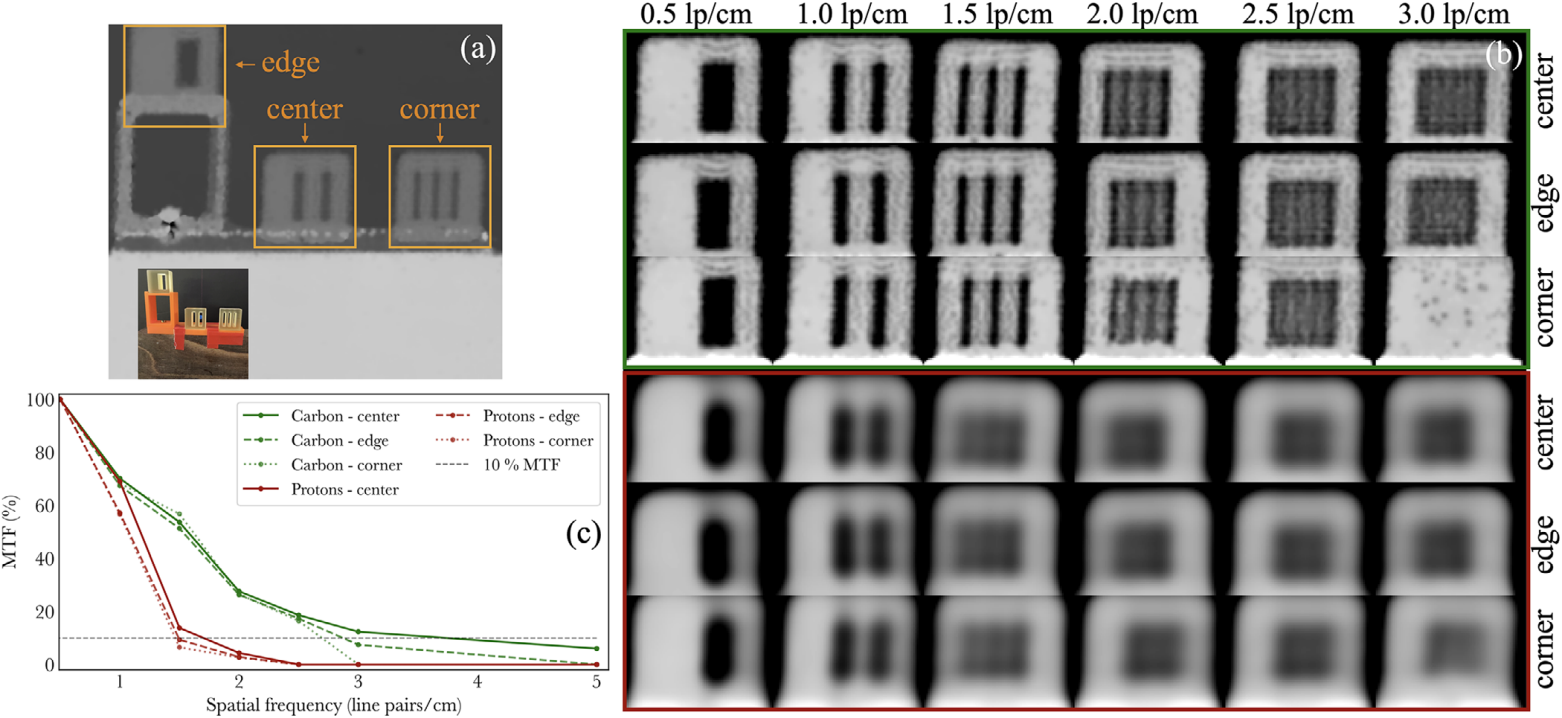
Evaluation of the spatial resolution for pRads and cRads for different locations in the imaging FOV. The locations are depicted in (a), while radiographs of each lp module are reported in (b). The corresponding modulation transfer functions associated with the modules of (b) are shown in (c). The dynamic range of pRads in (b) is from 10 to 12 cm. cRads, carbon ion radiographs; FOV, field of view; lp, line pair; pRads, proton radiographs.

General image quality was also evaluated using an anthropomorphic head phantom (Alderson head phantom, Radiology Support Devices Inc., Gardena, Ca), scanned in two different positions ‐ lateral to the beam propagation axis (lateral view), and facing the beam (front view). Both views are shown in Figure 5. For the front view, the beam energy was adjusted respectively to 200 MeV and 390 MeV/u for protons and carbon ions such that the beam could be transmitted through the thickest part of the phantom.

**FIGURE 4:**
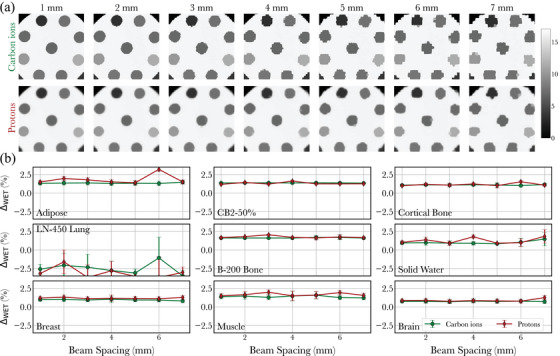
WET accuracy evaluated on tissue substitute inserts. (a) shows ion radiographs for both ion species as a function of beam spacing, while (b) reports the relative WET error in each plug as a function of spot spacing. WET, water equivalent thickness.

**FIGURE 5:**
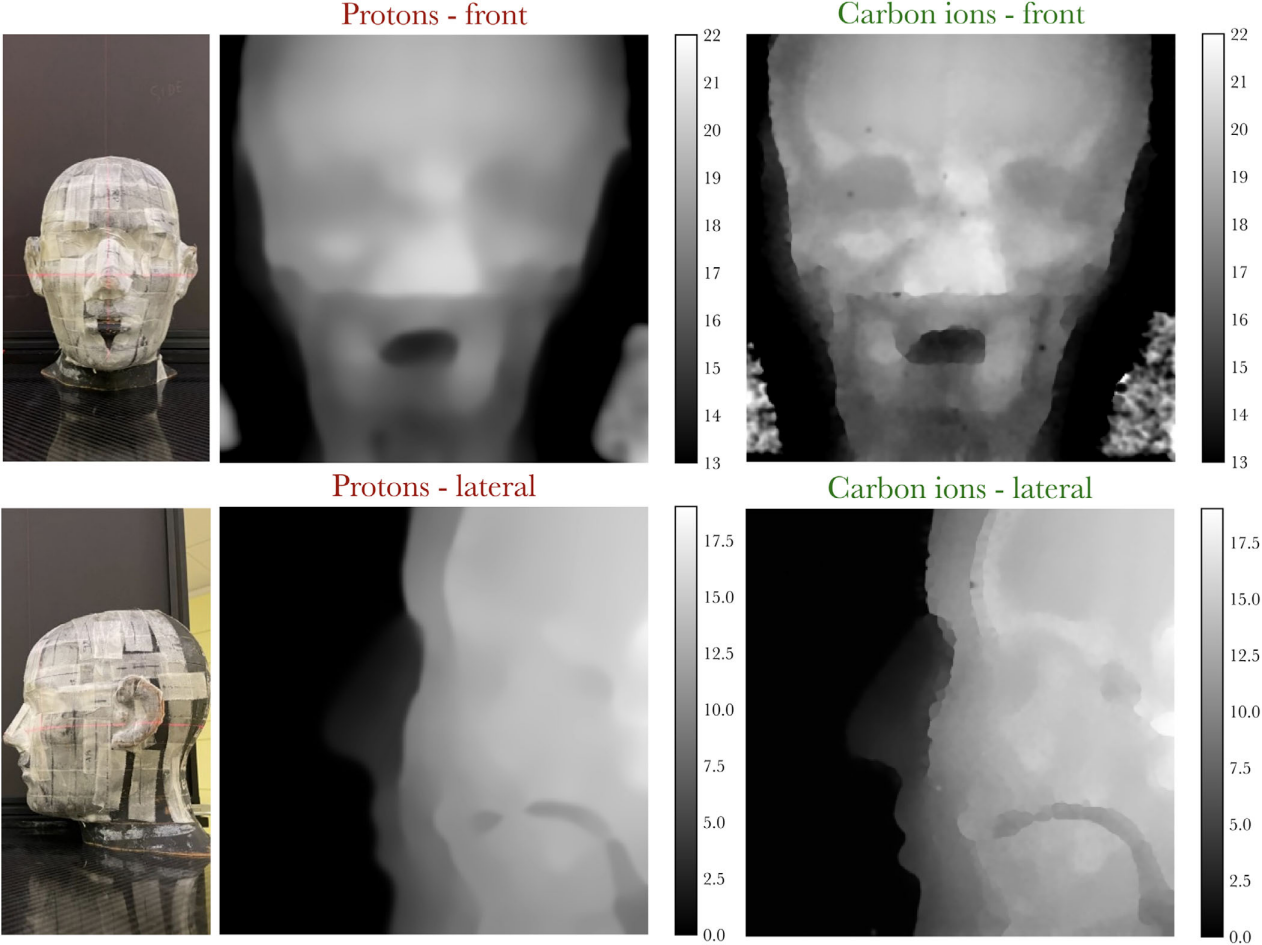
Photographs (first column) and ion radiographs (second and third columns) of two different views of an anthropomorphic head phantom. The reported intensities are WET values (cm). WET, water equivalent thickness.

The detection of low contrast objects was studied using a custom 3D printed low contrast module consisting of 20 small circular holes with depths of d = [1, 3, 5, and 8] mm and diameters of ϕ = [1, 2, 4, 7, and 10] mm. The phantom is shown in Figure 1e. The printer and material were the same as for the lp modules. As for the lp modules, a 10 cm slab of PMMA was placed proximal to the low contrast phantom.

Finally, to evaluate quantitative WET accuracy, nine tissue‐substitute Gammex 467 inserts (Gammex Inc., Middleton, WI) were scanned. The Gammex inserts were placed in a custom cylindrical holder made of PMMA, intended for calibrating head & neck CT scanning protocols at MIT. The phantom was positioned vertically such that the Gammex inserts were positioned parallel to the beam direction. The materials are listed in Figure 4b.

### 2.2 Raw data processing

Raw images obtained from CCD cameras were corrected for various optical artifacts to obtain geometrically consistent images. Camera distortion,^16^ optical vignetting,^17^ perspective, and refraction corrections were applied to each image, as described in earlier work.^14^, ^15^ Overall, the corrections interpolated the raw data such that all pencil beams appear as if they entered the scintillator at the face closest to the camera for which the correction is applied. WET maps were reconstructed from raw data following the 2D lateral method introduced in Simard et al.^14^ For each pencil beam, multiple candidate Bragg peaks are identified with a 2D peak‐finding routine, and the lateral positioning of the beams are inferred from the two matching perpendicular lateral images of the beam. The peak‐finding routine is based on applying the methodology of ref. [18] on integral depth dose signals obtained by summing the 2D images in the lateral dimension. More details on the peak‐finding routine can be found in section 2.3.2 of ref. [14]. WET maps are reconstructed via a weighted WET reprojection using a point spread function that takes into account MCS in the object and detector.

All iRads were reconstructed using the 22 801 pencil beams sampled every 1 mm, with the exception of the WET accuracy results shown in Figure 4, where the impact of beam spacing on WET accuracy was investigated. In that case, iRads of the Gammex inserts were reconstructed for beam spacings of 2–7 mm by sub‐sampling the 1 mm datasets.

### 2.3 Image quality quantification

Low contrast modules were used to evaluate the detection performance of low contrast objects of small sizes (1–10 mm diameter). Each hole with diameter ⌀ and depth d is characterized by its WET contrast $C_{\mathrm{WET}}={\mathrm{WET}}_{\mathrm{ref}}-{\mathrm{WET}}_{h}$, where ${\mathrm{WET}}_{\mathrm{ref}}$ is the average WET in the vicinity of a given hole and ${\mathrm{WET}}_{h}$ is the average WET inside the hole. For each hole in the phantom, both quantities are calculated using manually defined regions of interest. The values of $C_{\mathrm{WET}}$ obtained from pRads and cRads can be compared with the expected value of each hole assuming water equivalence of the 3D printed material; in that case, $C_{\mathrm{WET}}$ is equal to the hole depth d. This is reported in Figure 2e.

To estimate the spatial resolution from iRads, the modulation transfer function (MTF) was calculated from the set of reconstructed WET maps of lp modules, illustrated in Figure 3c. Briefly, a region which only includes the lps was manually cropped for each module shown in Figure 3c. Then, the 1D profile of the lps (representing spatial frequency ξ), $P_{\xi }\left(r\right)$, was obtained by averaging the cropped image in the vertical direction. The contrast at spatial frequency ξ is then defined as

(1) $$C\left(\xi \right)=\frac{\max\left(P_{\xi }\left(r\right)\right)-\min\left(P_{\xi }\left(r\right)\right)}{\max\left(P_{\xi }\left(r\right)\right)+\min\left(P_{\xi }\left(r\right)\right)}$$

The MTF is obtained as $C\left(\xi \right)/C\left(\xi_{0}\right)$, where $\xi_{0}$ is the lowest spatial frequency in the lp modules. A total of 6 MTFs, shown in Figure 3b, are constructed: one for each location in the imaging FOV (center, edge, corner) and ion species (protons, carbon ions). The resolution is the spatial frequency ξ at which the MTF drops to 10%, and was obtained via linear interpolation.

For quantitative WET accuracy, ground truth WET data for each Gammex insert, ${\mathrm{WET}}_{0}$, was obtained using a water column (PTW Peakfinder), as detailed in Witt et al.^19^ Estimated WET values, $\tilde{\mathrm{WET}}$, were obtained from the average inside a region of interest representing the inner 75% of each insert. The relative WET error in each plug, $\Delta_{\mathrm{WET}}=\frac{\tilde{\mathrm{WET}}-{\mathrm{WET}}_{0}}{{\mathrm{WET}}_{0}}$, is reported, along with the relative standard deviation in the insert.

## 3 RESULTS

Figure 2 shows the low contrast detectability performance of proton and carbon ion radiographs. For cRads, holes with a diameter as low as ⌀ = 4 mm can be identified (non‐zero value of $C_{\mathrm{WET}}$ in Figure 2e), although the contrast $C_{\mathrm{WET}}$ cannot be accurately recovered for this diameter (Figure 2e). Holes with a depth of up to d = 1 mm can be identified, albeit with large relative errors on $C_{\mathrm{WET}}$. Such errors are limited for holes of d = 3 mm. For pRads, while holes of up to 7 mm diameter can be identified, the contrast $C_{\mathrm{WET}}$ is systematically lower than for cRads and is consistently inaccurate across all holes.

A comparison of the resolution achievable with pRads and cRads is shown in Figure 3 and Table 1. cRads result in a systematically higher resolution than pRads for all tested locations in the FOV of the image; pRads are noticeably blurrier for all lp modules illustrated. For both ion species, the resolution is highest in the center of the image. The resolution, respectively drops by 21% and 11% from the center to the corner of the FOV with cRads and pRads, while there is another relative reduction of approximately 7% in resolution for both species when comparing the corner of the FOV and the edge. It is worth noting that the quoted spatial resolution values are for a WET of approximately 13 cm due to the addition of a 10 cm solid water slab in front of the inserts.

**TABLE 1:** Spatial resolution of integrated mode proton and carbon ion radiographs obtained from the modulation transfer functions of Figure 3b for three locations in the imaging FOV. The locations can be identified in Figure 3a.

	Resolution (lp/cm)	
Location in FOV	Protons	Carbon ions
Center	1.7	3.7
Corner	1.5	2.9
Edge	1.4	2.7

Abbreviations: FOV, field of view; lp, line pair.

WET accuracy, evaluated on tissue‐equivalent inserts, is reported in Figure 4, for each ion species and for various spot spacings, ranging from 1 to 7 mm. The root mean squared error (RMSE) on WET accuracy, calculated over all 9 inserts shown in Figure 4b and for a spot spacing of 1 mm, is similar for both ion species. For carbon ions and protons, the values are, respectively, 0.99 mm (1.43% relative) and 1.02 mm (1.59% relative). Quantitative accuracy is shown to vary slightly as a function of beam spacing, with the RMSE increasing to respectively, 1.05 mm and 1.14 mm for the reconstructed radiographs with a beam spacing of 7 mm. However, there are apparent artifacts that appear in the radiographs of Figure 4a obtained with larger spacings, which result in distorted inserts. Such artifacts are more apparent for cRads compared to pRads.

iRads of the anthropomorphic head phantom, illustrated in Figure 5, show improved contrast, especially around bony structures, for carbon ions. This includes, for instance, the skull in the lateral view as well as the brow ridge in the front view. pRads are found to be generally blurrier.

## 4 DISCUSSION

The image quality of integrated mode ion radiographs has been evaluated in this work. Overall, image quality is largely improved using carbon ions as opposed to proton beams, similarly to what has been reported in previous studies.^7^, ^12^ For integrated mode iRads and the reconstruction method used in this work,^14^ image quality is essentially a function of the beam spot size that crosses the structures of interest in the object. A wider beam can pass through more heterogeneous structures and result in important range mixing. Small WET differences can be difficult to resolve in large beams and result in lower contrast and resolution for lighter charged particles. The beam size after crossing various materials is noticeably larger for pRads as opposed to cRads, as can be identified in Figure 1b and c. This is explained by the larger initial beam size of the proton beam against the carbon ion beam (9.4 vs. 5.2 mm FWHM), and a larger contribution of multiple Coulomb scattering to the spread of the beam with depth, which is approximately 3.6 times higher for protons than carbon ion beams,^7^ for the same particle range. Notice that the limitation on the achievable focus at isocenter is dominated by the scattering in the nozzle and subsequent drift in air. Improved image quality may be achievable by placing the object as close to the beam exit window as possible.

The improved low contrast detectability of cRads can mainly be attributed to the smaller spot size scanning the object after traversing the various materials in the beam's path. The contrast‐detail curves of Figure 2 demonstrate that for objects with an overall WET of approximately 13 cm, cRads can resolve WET differences of up to 1 mm with objects with a diameter of 4 mm. With protons, while there is a quantifiable contrast difference for objects of 7 mm diameter, the actual objects cannot be practically resolved in the radiographs, illustrating that the studied objects are close to the resolution limit of pRads in the studied conditions. In that context, it becomes difficult to evaluate low contrast detection for small objects with pRads, as the impact of the limited resolution cannot be easily deconvolved from the results. Nonetheless, focusing on the largest objects with a diameter of 10 mm, it is apparent that pRads still present limited contrast, considering that the WET of the deepest hole (d = 8 mm) cannot be recovered.

Image resolution was approximately improved by a factor of two using cRads rather than pRads and is also a direct consequence of the larger beam size associated with pRads. This is consistent with single‐event imaging studies.^7^ There are no other reports of cRads spatial resolution for integrated mode iRads; however, the value of 3.7 lp/cm obtained with cRads is only slightly lower than the resolution of 4.9 lp/cm obtained for single event HeRads.^20^ We report that the resolution of integrated‐mode iRads is also position‐dependent in the FOV, as it monotonically decreases the further an object is from the center of the FOV. We mainly attribute this effect to beam divergence; while beams in the center of the FOV are mainly traveling in a straight direction, beams at the edge of the field can have a divergence angle of approximately $1^{\circ }$ in each direction towards the edge of the field. This may create additional range mixing effects and lead to a reduced spatial resolution. While the spatially‐variant spatial resolution is not ideal, it is a consequence of imaging large FOVs with entire pencil beams without any information on particle position. Our results suggest that smaller objects which require high spatial resolution should ideally be in the center part of the FOV to maximize the performance of integrated mode iRad devices.

WET accuracy is comparable for cRads and pRads, with an average error of 1 mm (1.5%) on the WET for both modalities. Our results are consistent with the conclusions of Kopp et al.^12^ who have reported that various species (protons, helium, and carbon ions) are almost equally capable of resolving reference WET values far from interfaces. The WET RMSEs reported in this work are generally lower than those reported in Kopp et al.,^12^ which are 2% for cRads and 2.6% for pRads using 6 tissue equivalent inserts. Compared to our previous work on pRads obtained with a similar scintillator setup, we obtained slightly better pRad WET accuracy ‐ 1.5% RMSE for this work, against 1.2% as reported in ref. [14]. We mainly attribute the small discrepancy in WET accuracy to a different set of materials scanned across studies.

All of the above results have been produced using a beam spacing of 1 mm. While this spacing produces high image quality for iRads, using a dense sampling leads to high dose and imaging time, which is incompatible with certain applications such as rapid fluoroscopic imaging for tumor tracking.^15^ In that context, it is relevant to evaluate image quality metrics as a function of spot spacing. The results of Figure 4 illustrate that WET accuracy in homogeneous regions can be preserved for spot spacing of up to 7 mm, which reduces the number of PBs by a factor of ∼50, hence limiting dose and imaging time. However, a coarser beam spacing leads to important distortions in the reconstructed WET maps, and is generally a limitation of integrated mode imaging. In general, we have not reported spatial resolution and low contrast detectability for coarser samplings, as the position of the beams close to the edge may have an important effect on such metrics; we leave this to future studies. It is also found that sampling artifacts are reduced with pRads compared to cRads. This is a consequence of the larger spot size of proton beams, which can better sample the geometry with a coarser spacing, albeit at the cost of reduced contrast and resolution.

Based on a visual assessment of the reconstructed anthropomorphic head phantom of Figure 5, the use of heavier ions for radiographic imaging can lead to largely improved image quality and allow a clearer identification of anatomical features compared to protons. Overall, the results of this work are useful to demonstrate the potential utility of image guidance with iRads in heavy ion facilities. Finally, the main limitation of this work is that reported image quality metrics are valid in the context of the imaging conditions used. This includes the beam spacing, the beam spot sizes, energies, and the average WET of the analyzed objects, as the contribution of MCS to beam spread is dependent on the traversed WET. It is also specific to the currently used scintillator‐based detector, and WET reconstruction technique that leverages two camera views, which influences image quality metrics.^14^

The imaging dose in this study was not optimized, but set to a very high level due to limitations with the imaging setup at MIT. We estimated the dose to water through forward calculations of the irradiated plans using the research treatment planning software TRiP98.^21^ For the $15.1\times 15.1\;{\text{cm}}^{2}$ fields with 1 mm spot spacing, the physical dose for both protons and carbon ion plans was estimated at 0.95–1.05 Gy, which is much larger than what would eventually be used for patients. Imaging dose scales with beam spacing ‐ for instance, the dose was reduced to ∼40 mGy for 5 mm spot spacing, which still produces acceptable image quality and quantitative accuracy. Nevertheless, the dose level per spot was overall high, and was imposed by limitations related to the use of the detector at MIT for the first time. First, for a single pencil beam delivery, the time window during which the beam is delivered could not be well aligned with the window during which the cameras are integrating. As a result, only a fraction of the delivered protons will contribute to the signal, and the dose per beam was increased to obtain satisfactory signal. We believe that this mismatch is due to a delay or an inconsistency between the next‐point trigger signal and the start of image acquisition, but lacked experimental time at MIT to fully investigate the issue; this is a limitation of the setup at MIT that we plan to investigate in further studies. In addition to the above mismatch, we could not optimize (1) the number of particles per spot and (2) the gain for each CCD camera, such that we would obtain signal with the lowest possible dose. Achieving reasonable imaging dose is feasible by fixing the above limitations, but requires additional beam time at MIT. The high dose obtained during this experiment is, however, not a limitation of our detector. In previous work, we have shown that high quality images can be obtained in principles by reducing the imaging dose by two orders of magnitude,^14^ which would reduce imaging dose to approximately 10 mGy. In that context, the dose is comparable to other integrated mode studies.^12^, ^22^

## 5 CONCLUSION

In this study, we reported the first experimental investigation of extensive image quality metrics for integrated mode carbon ion imaging. Such metrics include spatial resolution, low contrast object detectability, and quantitative WET accuracy. The metrics were obtained by using a scintillator‐based detector to acquire radiographs of custom 3D printed objects for spatial resolution and low contrast evaluation, as well as tissue equivalent inserts for WET accuracy. The overall image quality was also assessed using an anthropomorphic head phantom. Image quality metrics were compared with integrated mode proton radiographs and were found to be systematically improved for carbon ions. For carbon ion radiographs, the resolution was found to be 3.7 lp/cm in the center of the field of view, while small objects with a diameter of 7 mm and 1 mm depth could be resolved. Spatial resolution varied across the imaging FOV, and that quantitative WET accuracy was preserved when beam spacing is reduced from 1 to 7 mm. Overall, proton radiographs were still shown to preserve high WET accuracy and deliver acceptable image quality. Given the broader accessibility and adoption of proton beam therapy as well as the higher image quality achievable with cRads, both cRads and pRads should be investigated for usage in adaptive workflows in ion beam therapy.

## CONFLICT OF INTEREST STATEMENT

The authors declare no conflicts of interest.
